# Novel pseudo-aspartic peptidase from the midgut of the tick *Rhipicephalus microplus*

**DOI:** 10.1038/s41598-018-36849-4

**Published:** 2019-01-24

**Authors:** S. Lu, L. F. Parizi, R. J. S. Torquato, I. S. Vaz Junior, A. S. Tanaka

**Affiliations:** 1 Department of Biochemistry, Federal University of Sao Paulo (UNIFESP), SP, Brazil; 2 Center of Biotechnology, Federal University of Rio Grande do Sul (UFRGS), RS, Brazil; 3 School of Veterinary, Federal University of Rio Grande do Sul (UFRGS), RS, Brazil; 4 National Institute of Science and Technology in Molecular Entomology (INTC-EM), RJ, Brazil

## Abstract

The characterization of *Rhipicephalus microplus* tick physiology can support efforts to develop and improve the efficiency of control methods. A sequence containing a domain with similarity to one derived from the aspartic peptidase family was isolated from the midgut of engorged female *R*. *microplus*. The lack of the second catalytic aspartic acid residue suggest that it may be a pseudo*-*aspartic peptidase, and it was named RmPAP. In this work we confirm the lack of proteolytic activity of RmPAP and investigate it’s non-proteolytic interaction with bovine hemoglobin by Surface Plasmon Resonance and phage display. Moreover we carried out RNAi interference and artificial feeding of ticks with anti-RmPAP antibodies to assess it’s possible biological role, although no changes were observed in the biological parameters evaluated. Overall, we hypothesize that RmPAP may act as a carrier of hemoglobin/heme between the tick midgut and the ovaries.

## Introduction

Ticks are recognized worldwide as major vectors for several pathogens, including arboviruses, rickettsiae, spirochaetes and parasitic protozoa, that can infect humans and livestock animals^1^. *Rhipicephalus microplus* is an exclusive bovine ectoparasite that is responsible for losses, estimated in 7.11 USD billions in Brazil^2^. Tick control traditionally involves the use of acaricides, which has several drawbacks, including environmental contamination and the development of resistant populations^3,4^. Vaccination could be used as an alternative method of control, but the discovery of protective antigens remains a challenge^5^.

Aspartic peptidases are characterized by the presence of two aspartic catalytic residues^6^ and, in ticks, they are mostly associated with protein degradation^7,8^. In *R*. *microplus*, three aspartic peptidases have been identified and characterized. The Tick Heme-Binding Aspartic Proteinase (THAP) is purified from eggs and is able to bind to heme, which can modulate its proteolytic activity towards hemoglobin and vitellin^9,10^. The *Boophilus microplus* aspartic peptidase (BmAP) is found in the tick midgut, and its activity towards bovine hemoglobin has been demonstrated. Moreover, the degradation of hemoglobin by BmAP appears to produce hemocidins that can play a role in pathogen control^11^. Finally, the native *Boophilus* Yolk-pro cathepsin (BYC) is purified from tick eggs and is capable of degrading hemoglobin and vitellin, despite the lack of a second aspartic catalytic residue^12–14^.

Enzymes are currently classified into families based on their inclusion of catalytic residues, the reaction that they catalyze and their molecular structure archetype^15^. Interestingly, an increasing number of sequences that are similar to enzymes but lack key catalytic residues have been identified^16,17^ and are currently known as “dead enzymes” or “pseudoenzymes”. Pseudoenzymes appear to be widely conserved and have been found in more than 20 different protein families among several organisms^18,19^. Although there has been no formal analysis of the evolution of pseudoenzymes to date, it is believed that such molecules emerge via gene duplication followed by the mutation of the key residues in the cognate enzyme^20,21^. Despite the loss of their characteristic enzymatic activity, pseudoenzymes have emerged as important proteins that act as allosteric regulators of active enzymes^22^, signal integrators^23,24^ and as regulators of protein trafficking^25^. Most biochemical studies of pseudoenzymes have been carried out in *Drosophila* or mammals^26–28^. In this study, we characterized a novel pseudo-aspartic peptidase from the tick *R*. *microplus*, RmPAP that lacks the second catalytic aspartic acid residue. Data obtained from Surface Plasmon Resonance and phage display experiments suggest that RmPAP may act as a hemoglobin carrier instead of a digestive enzyme.

## Results

### Amplification and cloning of the RmPAP ORF

**Figure 1:**
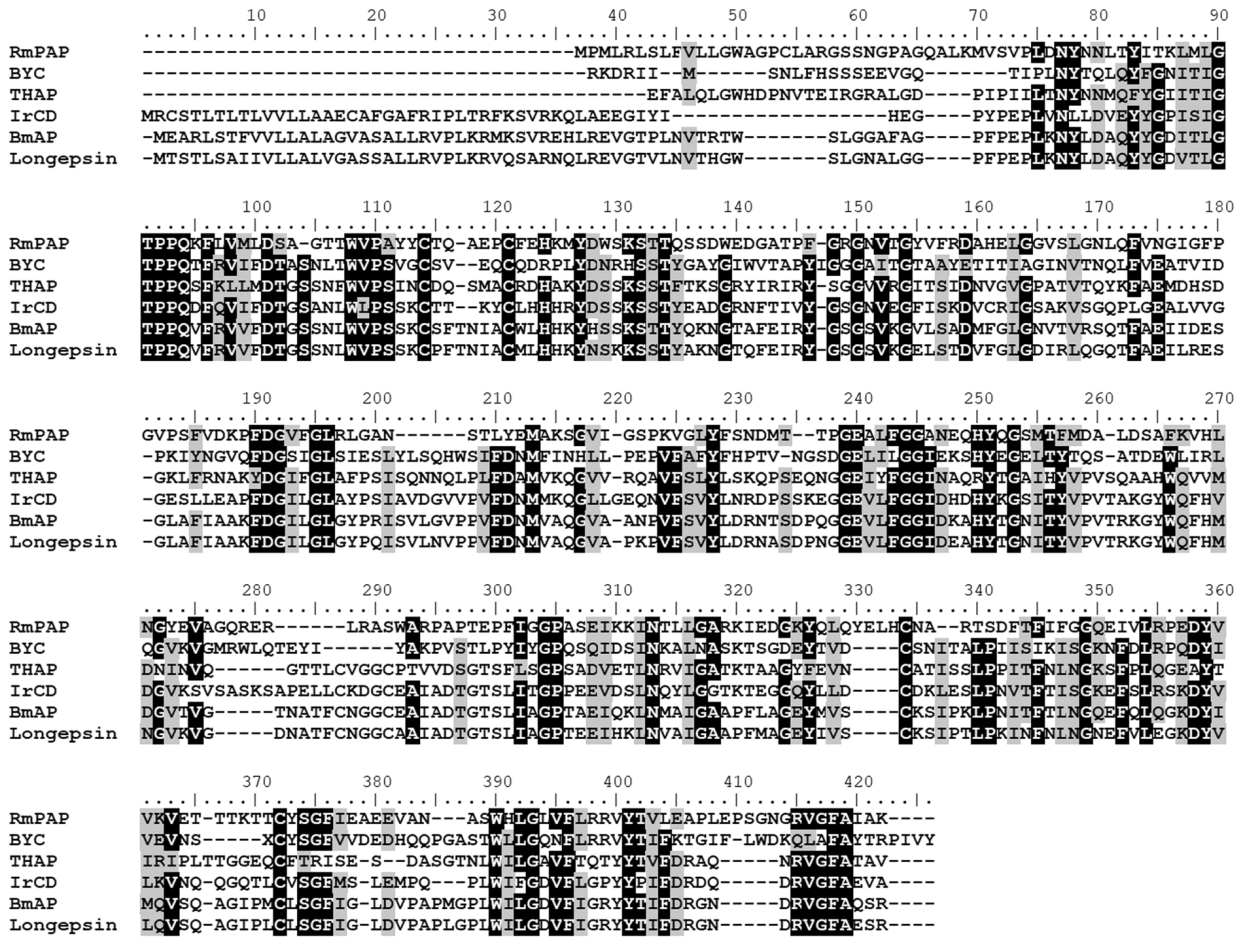
Amino acid alignment of RmPAP (GenBank: MH427522) with aspartic peptidases from other ticks. BYC (GenBank: AAX76981.1), THAP (GenBank: AAG00993.1) and BmAP (GenBank: ACP21315.1) are from *R*. *microplus*, IrCD (GenBank: ABO26561.1) is from *Ixodes ricinus* and Logepsin (GenBank: BAE53722.1) is from *Haemaphysalis longicornis*. Identical residues are in black while similar residues are in gray. Arrows indicate the catalytic Asp residues.

The complete nucleotide sequence (Sup. Figure 1) of the *R*. *microplus* pseudo-aspartic peptidase was amplified from the midgut of the engorged females. The amino acid sequence derived from the translation of RmPAP mRNA revealed the presence of a putative signal peptide (M^1^ – A^20^) and the lack of a second catalytic Asp residue (Fig. 1). The mature protein (R^21^ – K^361^) had a theoretical pI of 5.76 and a molecular weight of 37.3 kDa. A mutant form (Pro^242^ > Asp^242^) was generated to restore the proteolytic activity (Sup. Figure 2).

### Expression and localization of RmPAP in *R. microplus* tissues

**Figure 2:**
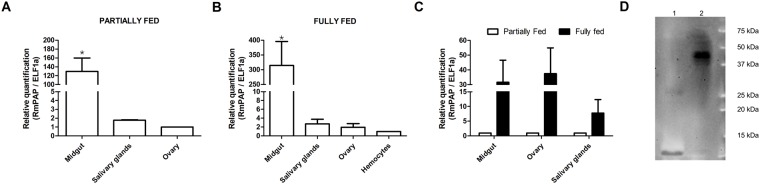
Localization of RmPAP mRNA in *R*. *microplus* tissues as detected by real-time PCR using cDNA preparations from partially (**A**) and fully (**B**) fed female ticks. (**C**) Modulation of the level of RmPAP transcripts during the engorgement period. (**D**) Western blot of proteins from the (1) midguts and (2) ovaries of fully-fed *R*. *microplus* ticks. The error bars represent the standard error of the mean from three independent experiments. *p = 0.03 as determined using the Kruskal-Wallis test with Bonferroni’s multiple comparison post hoc test.

RmPAP expression was observed mainly in the midgut of partially (Fig. 2A) and fully fed females (Fig. 2B). The comparison of the levels of expression between partially and fully fed females demonstrated that RmPAP expression was up-regulated in three tissues that were analyzed, including the midgut (30-fold greater), ovary (35-fold greater) and salivary glands (8-fold greater) (Fig. 2C). Western blot assays using purified anti-RmPAP antibodies (Sup. Figure 3) revealed the presence of a minor 25 kDa product in the midgut and a major product of approximately 40 kDa in the ovaries of engorged ticks (Fig. 2D).

### Expression and purification of recombinant RmPAP_WT_ and RmPAP_MUT_

**Figure 3:**
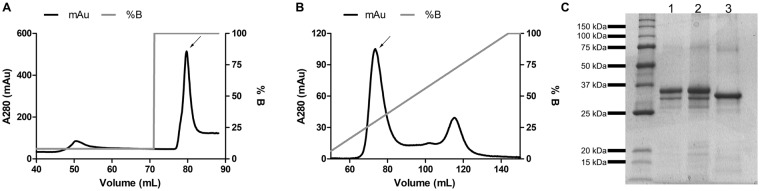
Purification of recombinant RmPAP using (**A**) affinity-chromatography with a Ni-NTA resin, with a two-step elution in 40 mM and 400 mM imidazol. (**B**) Ionic exchange chromatography with HiTrap Q resin, with elution being conducted with a crescent linear gradient in 100 mM Tris-HCl (pH 8.0) containing 8.0 M urea and 1.0 M NaCl. Arrows indicate the elution fractions containing the recombinant protein. (**C**) SDS-PAGE of purified RmPAP_WT_ (1) before and (2) after the refolding process and RmPAP_MUT_ (3) after refolding.

Protein expression was tested in different bacterial strains with a wide range of temperatures, IPTG concentrations and induction times, but at all conditions tested, both recombinant proteins were obtained in insoluble form and become soluble only in the presence of 8.0 M urea (data not shown). After protein purification (Fig. 3A and B), a major protein product of 36 kDa was observed (Fig. 3C), and after refolding RmPAP_WT_ was observed to have a mass of 36 kDa while RmPAP_MUT_ was observed to have a mass of 32 kDa (Fig. 3C).

### Interaction of RmPAP with bovine hemoglobin

**Figure 4:**
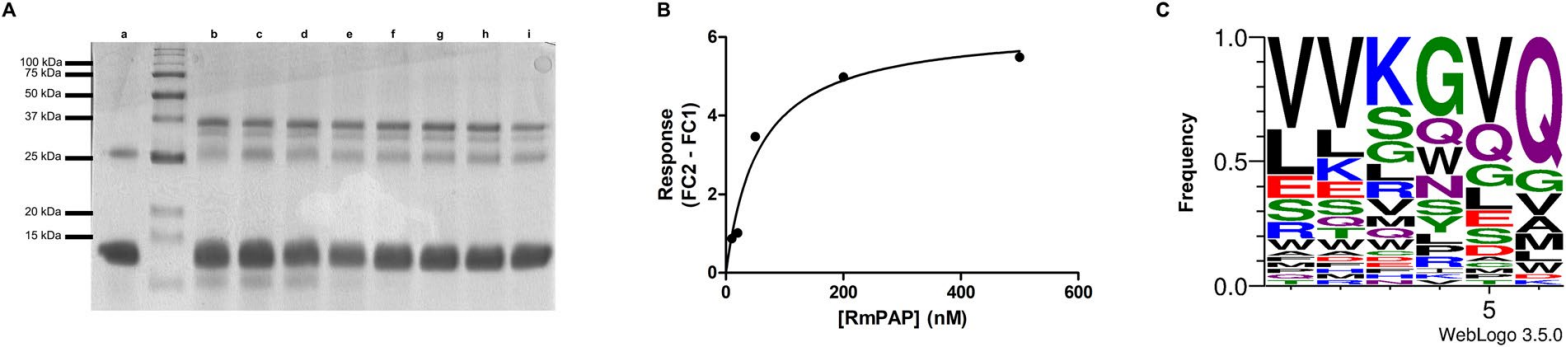
RmPAP interaction with bovine hemoglobin. (**A**) SDS-PAGE (15%) of (a) bovine hemoglobin and RmPAP_WT_ and bovine hemoglobin at pH 2.5 (b), 3.0 (c), 3.5 (d), 4.0 (e), 4.5 (f), 5.0 (g), 5.5 (h) and 6.0 (i). (**B**) The response (Fc2 – Fc1) was plotted against the concentration of recombinant RmPAP_WT_; the curve was fitted using steady-state kinetics in the Biacore T200 evaluation software. (**C**) Graphical representation of the frequency of the translated peptides at the six mutated positions among the 40 randomly selected clones derived from phage display selection with recombinant RmPAP_WT_.

After refolding, wild-type RmPAP showed no proteolytic activity towards bovine hemoglobin (Fig. 4A), while the site-directed mutation (Asp^242^) was demonstrated to restore proteolytic activity (Sup. Figure 4). Preliminary data from native-PAGE using RmPAP_WT_ and bovine hemoglobin revealed a possible interaction between the two molecules (Sup. Figure 5A). To further investigate this interaction, SPR experiments were conducted, which found a strong affinity (K_D_ = 3.35 × 10^−8^ M) of RmPAP_WT_ for bovine hemoglobin (Fig. 4B). To assess the specificity of rRmPAP, a screening against a hexapeptide library was performed using phage display. After three rounds of selection against rRmPAP (Sup. Table 2), the phages were sequenced and a high prevalence (21%) of the V-V-K-G/E-Q peptide was found (Fig. 4C).

### Determination of the biological effects of RNA interference and anti-RmPAP antibodies

**Figure 5:**
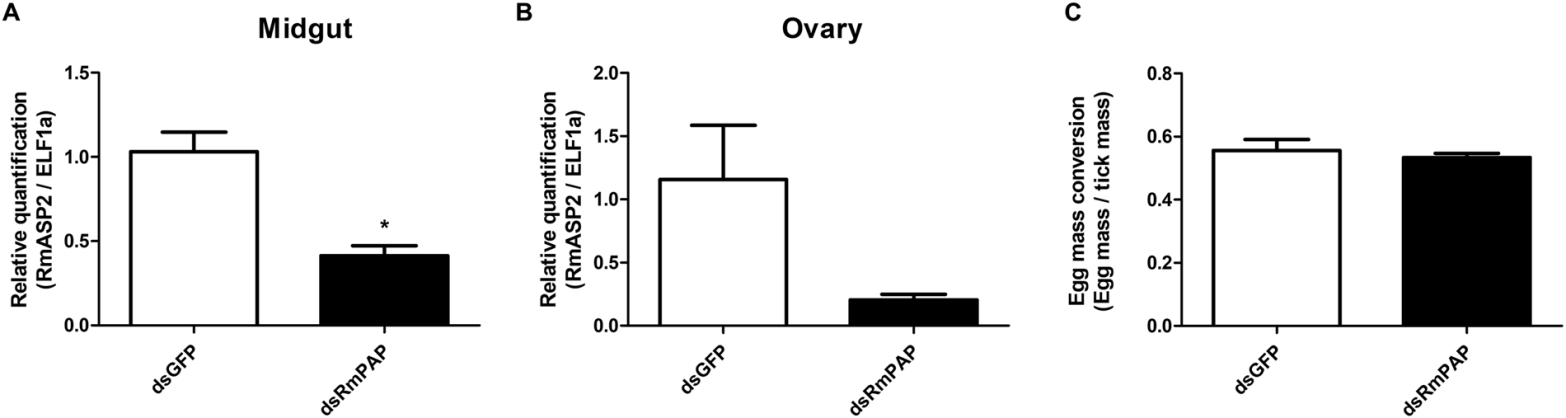
RmPAP knockdown was confirmed by qPCR in the (**A**) midgut and (**B**) ovaries of engorged *R*. *microplus* females 48 hours post-injection with dsRNA. (**C**) Effects of RmPAP gene silencing on the total egg mass. The relative quantification was calculated using the 2^−ΔΔCt^ method with ELF1α as the endogenous control and dsGFP as the non-related control. *p = 0.0022 as determined using the Mann-Whitney test.

**Figure 6:**
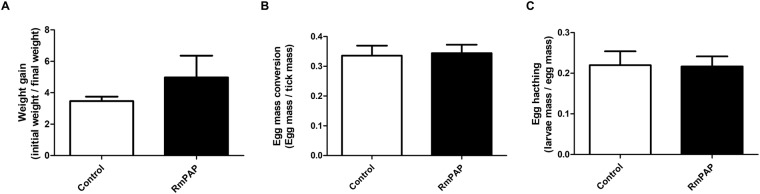
Artificial feeding of partially-engorged *R*. *microplus* females. Differences in (**A**) weight gain after the artificial feeding, (**B**) the rate of egg laying and (**C**) the rate of egg hatching in ticks fed control antibodies or anti-RmPAP antibodies (n = 30 ticks per group). Statistical analysis was performed using Student’s t-test, which found no statistically significant differences.

To verify the possible role of RmPAP in ovary physiology, the inhibition of its activity was carried out during artificial feeding using both RNA interference and anti-RmPAP antibodies. Gene silencing was confirmed by qPCR in both the midgut (60% reduction) and ovaries (80% reduction) 48 hours post-injection (hpi) (Fig. 5A and B), although no difference in egg mass was observed (Fig. 5C). Likewise, the biological parameters that were assessed post-artificial feeding, such as weight gain (Fig. 6A), total egg mass (Fig. 6B) and egg hatching (Fig. 6C), also presented no significant differences.

## Discussion

Aspartic peptidases are characterized by the presence of two aspartic acid residues that are required for their catalytic activity^6^ and, in ticks, they are typically located in the midgut^29,30^ and involved in protein degradation^7,11^. The advances in sequencing technologies shred light into a new class of molecules known as pseudoenzymes. Pseudoenzymes are proteins with high similarity to functioning enzymes but that lack residues that are key to their catalytic activity^18,31^. Despite the absence of catalytic activity, pseudoenzymes have emerged as important regulators of different physiological processes. In this report, we describe the characterization of the first pseudo-aspartic peptidase from the tick *R*. *microplus* and its possible functioning as a hemoglobin carrier.

The domain analysis of the deduced RmPAP amino acid sequence reveals its similarity to other conserved aspartic peptidases, while BLASTp revealed its similarities to other aspartic peptidases in ticks (Fig. 1). However, RmPAP lacks the second catalytic aspartic acid residue suggesting the absence of proteolytic activity.

RmPAP is a major transcript in the midgut of both partially and fully fed females and appears to be up-regulated as digestion progresses, although the native protein was observed only in the ovaries of fully engorged females. A similar expression profile was observed for other aspartic peptidases, such as BYC and THAP. Both of the enzymes were shown to be expressed in extra-ovarian tissues (midgut and fat body), but the native protein was detected only in the hemolymph and ovaries^9,10,12^. It suggests that the enzymes may be produced and secreted to the hemolymph and carried to the ovaries, where they are accumulated. Since the same pattern was observed for RmPAP, it’s tempting to suggest the same mechanism and is thus more relevant to ovary physiology than blood digestion.

Pseudoenzymes are a newly discovered group of molecules that have been identified with advances in sequencing techniques. These molecules’ sequences usually resemble that of an archetypical enzyme but lack enzymatic activity^32^ due to mutations that disrupt or occlude the original catalytic site^18,31^. It’s important to note that mutation of the catalytic residues is not proof of the absence of enzymatic activity. In *Plasmodium falciparum*, a protein containing an aspartic peptidase domain that also lacked the second catalytic Asp residue showed proteolytic activity towards hemoglobin, and a His residue was found to play an important role in this activity^33,34^. The aspartic peptidase from *R*. *microplus*, BYC, also lacks one of the Asp residues but is still able to process bovine hemoglobin^14^, indicating the existence of a-yet-to-be-discovered enzymatic mechanism. Mature RmPAP was obtained from bacterial inclusion bodies and, after purification and refolding, it maintained its 37 kDa protein size (Fig. 3 – Lane 2). Moreover, when incubated with bovine hemoglobin, RmPAP_WT_ did not display proteolytic activity (Fig. 4A). To verify if the lack of RmPAP_WT_ activity was due to the absence of the second Asp residue, a mutant (Pro^242^ > Asp^242^) was generated and processed using the same conditions. After refolding, RmPAP_MUT_ appeared as a 32 kDa protein band, suggesting that N-terminal processing by auto-activation occur and incubation with bovine hemoglobin resulted in its degradation (Sup. Figure 4). Overall, these data strongly suggest that RmPAP_WT_ lacks the expected proteolytic activity and can be considered a pseudoenzyme.

It is believed that pseudoenzymes originate from gene duplications of active enzymes followed the accumulation of mutations that render them inactive; despite this, they maintain some of the functional characteristics from their ancestors. Preliminary analysis using native-PAGE (Sup. Figure 5A) suggested that RmPAP_WT_ could alter the migration profile of bovine hemoglobin, and SPR data revealed a strong affinity (K_D_ = 3.35 × 10^−8^ M) (Fig. 4B) between the two molecules. This verified that RmPAP could bind, in a non-proteolytic fashion, to bovine hemoglobin, most likely due the presence of a conserved structural feature typical of aspartic peptidases. Since the post-refolding protein yield was inadequate for structure studies, as well as the feasibility of high-throughput screening with phage display, we decided to investigate the possible binding site(s) and specificity of RmPAP_WT_ using a hexapeptide phage display library^35^. After two rounds of selection, an enrichment of 34-fold was observed (Sup. Table 2), indicating that specific phages were selected in the presence of RmPAP_WT_. The analysis of the selected phages reveals that a high proportion of the peptide V-V-K-G-V/E-Q (Fig. 4C). Interestingly, the VKG peptide can also be found in an exposed segment of the bovine hemoglobin chain A α-helix (Sup. Figure 5B), which could serve as a possible binding site for RmPAP_WT_.

To assess the possible role of RmPAP in ovary physiology, both gene silencing through RNA interference (Fig. 5) and the artificial feeding of partially-fed females with anti-RmPAP_WT_ antibodies (Fig. 6) was conducted. No differences were observed in the biological parameters that were evaluated. It suggests that the level of RmPAP was expressed before RNAi treatment and it was enough to maintain its biological functioning and/or that it has a low protein turnover rate, which would work to conceal the effects of RNAi silencing that were observed in other RNA interference experiments^36^. Moreover, bioinformatics analysis of the *R*. *microplus* transcriptome (SRA accession numbers: SRX484287, SRX484284, SRX484280 and SRX484277) revealed the presence of other contigs that are similar to RmPAP and lack the second aspartic catalytic residue (data not shown). Therefore, it’s tempting to hypothesize that the physiological activity of RmPAP may be compensated by these other molecules and that the lack of any one of these molecules may not suffice to produce a distinctive phenotype. Nevertheless, further studies are necessary to characterize the physiological roles of RmPAP.

In this study, we investigated a novel pseudo-aspartic peptidase from *R*. *microplus* females and found that RmPAP was able to bind to bovine hemoglobin in a non-proteolytic fashion with its possible binding site characterized. The high expression of RmPAP was observed in the midgut of both partially and fully fed females, although the protein appeared to accumulate in the ovary. Bearing those data in mind, we hypothesized that RmPAP may act as hemoglobin/heme carrier between the midgut and the ovaries and thereby contribute to ovary maturation.

## Materials and Methods

*Rhipicephalus microplus* Porto Alegre strain was reared on Hereford calves (*Bos taurus taurus*) in the Faculdade de Veterinaria, Universidade Federal do Rio Grande do Sul (UFRGS), Porto Alegre, RS, Brazil. This study was conducted according to the ethical and methodological guidelines of the International and National Directives and Norms by the Ethics Commission of animal use – CEUA – UFRGS.

### RNA extraction and cDNA synthesis

The ticks were washed with 70% ethanol followed by ultrapure water and dissected. The midgut, ovary, salivary glands and hemocytes were collected and added to Trizol (Invitrogen, CA, USA). RNA exctration was conducted according to the manufacturer’s instructions. The RNA was treated with DNAse I (Fermentas, Vilnius, LT) for 1 h at 37 °C and 1 μg was used for cDNA synthesis using the Improm-II Reverse Transcription System (Promega, WI, USA).

### Amplification and cloning of the RmPAP ORF

Primers (Sup. Table 1) containing the Xho I (sense) and Bpu1102I (anti-sense) restriction sites were designed based on the RmPAP_WT_ (*Rhipicephalus microplus* pseudo-aspartic peptidase wild type) nucleotide sequence (Sup. Figure 1), obtained from a *R*. *microplus* transcritpome (SRA SRX484287, SRX484284, SRX484280 and SRX484277). PCR was performed using 1 μL of midgut cDNA, 100 μM dNTPs, 1.5 mM MgCl_2_, 5 U Taq DNA polymerase (Sinapse, SP, BR) and 25 pmol of each primer. The reactions were subject to an initial denaturation at 94 °C for 10 min followed by 25 cycles of 94 °C – 30 s, 55 °C – 60 s, and 72 °C – 60 s with a final extension at 72 °C for 10 min. The PCR products were analyzed using a 1% agarose gel and purified with the QIAEXII extraction kit (QIAGEN, Hilden, DE). The purified PCR products were later cloned into a pET14b vector containing a N-terminal His tag.

### Primary structure analysis

A domain search was conducted using PFAM (https://pfam.xfam.org/)^37^ and the signal peptide was identified using SignalP 4.1 (http://www.cbs.dtu.dk/services/SignalP/)^38^. The theoretical molecular weight and pI were estimated by the Compute pI/MW tool (https://web.expasy.org/compute_pi/)^39^. The sequence alignment was performed using Clustal Omega (https://www.ebi.ac.uk/Tools/msa/clustalo/)^40^ and edited with the BioEdit software.

### Site-directed mutagenesis

The mutation of Pro^242^ to Asp^242^ was conducted by PCR. The first two reactions were performed using the RmPAP.FW/RmPAP.MUT.RV and RmPAP.RV/RmPAP.MUT.FW primers (Sup. Table 1). The PCR product was analyzed in 1% agarose gel and purified with the QIAEX II extraction kit (QIAGEN, Hilden, DE). The purified DNAs were mixed (1:1) and used as the template for a second PCR using the RmPAP.FW and RmPAP.RV primers. The resulting PCR product was purified and cloned into the pET14b vector.

### RmPAP expression and purification

The recombinant RmPAP_WT_ and RmPAP_MUT_ proteins were expressed in the *Escherichia coli* BL21 plys S at 37 °C with IPTG (1 mM). After 16 hours of induction, the culture was centrifuged (10 min, 3000 × g, 4 °C) and the cells were resuspended in 50 mM Tris-HCl (pH 8.0). Bacterial lysis was conducted by 3 cycles of French press. The samples were centrifuged (20 min, 12.000 × g, 4 °C), the supernatant collected, and the pellet washed with 50 mM Tris-HCl (pH 8.0) containing urea (2, 4, 6, and 8.0 M). Protein purification was performed in the presence of 8.0 M urea by affinity chromatography with Ni-NTA followed by ionic exchange chromatography with HiTrap Q resin. Protein refolding was performed in the presence of 25 mM Tris-HCl (pH 7.5), 0.4 M L-arginine, 0.15 M NaCl and 1 mM β-mercaptoethanol by dialysis against buffers containing: (a) 4 M urea and 0.4 M L-arginine for 3 h, (b) 2 M urea and 0.4 M L-arginine for 3 h, (c) 0.4 M L-arginine for 3 h and (d) plain buffer overnight at 4 °C as described in^30^.

### Real time PCR

Reactions were prepared with 6 µL of SYBR Green PCR Master Mix (Applied Biosystems, Warrington, UK), 1 µL of a five-fold dilution of the cDNA preparation, 200 nM of the qRmPAP primers (Sup. Table 1) and ELF1α (endogenous control^41^). The samples were subjected to 40 cycles (95 °C – 1 min, 60 °C – 1 min and 72 °C – 1 min) in a 7500 Fast Real Time PCR System (Applied Biosystems). Three independent experiments were performed, and the relative quantification was determined by the 2^−ΔΔCt^ method^42^.

### Western blot analysis

Protein was extracted from the midgut and ovaries of engorged *R*. *microplus* females using Trizol reagent (Invitrogen, CA, USA) according to the manufacturer’s instructions, and 10 µg of the total protein was separated using SDS-PAGE (12%). Proteins were transfer to a PVDF membrane using a Mini Trans-Blot Cell system (BioRad) for 1 h at 15 V. After transfer, the membrane was incubated for 2 h in blocking solution (PBS containing 0.1% Tween – PBS-T - and 5% skim milk) at room temperature, followed by incubation with purified anti-RmPAP antibody, diluted 1:10 in blocking solution, overnight at 4 °C (Sup. Method 1). The PVDF membrane was then washed 3 times with a PBS-T 0.1% and incubated with anti-rabbit IgG conjugated with peroxidase (1:5000) in blocking solution. After 2 h of incubation, the SuperSignal West Pico Chemiluminescent substrate (Pierce, IL, USA) was added and the membrane incubated for 10 min at room temperature. Imaging was performed using the MR-ChemBis 3.2 (DNR Bio-imaging System) by exposing the membrane to UV light for 3 min.

### Determination of RmPAP activity towards bovine hemoglobin

Refolded RmPAP_WT_ and RmPAP_MUT_ (2.0 µg) were incubated with bovine hemoglobin (5 µg) in 50 mM phosphate-citrate buffer (pH 2.5–6.0) for 4 h at 37 °C and analyzed using SDS-PAGE (15%). The binding of RmPAP_WT_ to bovine hemoglobin was measured by surface plasmon resonance (SPR) using a Biacore T-200 system. Bovine hemoglobin (2000 RFU) was immobilized on a CM5 series chip (FC 2) in acetate buffer (pH 5.5), while BSA was immobilized in FC 1. SPR experiments were conducted by injecting increasing concentrations of RmPAP_WT_ (10 mM phosphate-citrate buffer, pH 4, with 0.15 M NaCl) at 20 µL/min, with association and dissociation times of 300 sec and 900 sec, respectively. The equilibrium constant was determined by plotting the intensity of the steady-state response (FC2 – FC1) against the RmPAP concentration using the Biacore T200 evaluation software (GE Healthcare).

### Peptide library screening using the phage display system

A hexapeptide library^35^ was used to determine recombinant RmPAP_WT_ specificity. *E*. *coli* TG1-transformed cells were grown in 2YT medium containing ampicillin (200 μg/mL) and 2% glucose until the OD_550_ reached 0.5–0.7. The helper phage M13K07 was added at a multiplicity of infection of 50 and the medium replaced with 2YT containing ampicillin (200 μg/mL) and kanamycin (50 μg/mL). After 16 h of incubation at 37 °C, the fusion phage particles were screened with recombinant RmPAP_WT_. RmPAP_WT_ was adsorbed to a 96-well plate overnight at room temperature following blocking for 2 h at room temperature with PBS-T 0.005% (pH 7.4) and 2% BSA. Entry phages, pre-incubated with blocking solution (1:1), were added and incubated for 1.5 h at 30 °C following 10 washes with PBS-T 0.1% (pH 7.4). The elution was performed with 0.2 M KCl (pH 2.0) followed by neutralization with 1.0 M Tris-HCl (pH 8.0). The eluted phages were then used for *E*. *coli* TG1 transfection and subsequent amplification and titration. After 3 rounds of selection, 40 phagemids were randomly selected and sequenced. The translated peptides were represented using the WebLogo tool^43^.

### Silencing of the RmPAP gene via RNA interference

Double-stranded RmPAP RNA (dsRmPAP) was synthesized using the T7 Ribomax Express System (Promega, WI, USA). Engorged *R*. *microplus* females were injected with 4.0 μg of dsRmPAP or dsGFP and dissected 48 h post-injection (10 ticks). The midgut and ovaries of dissected ticks were added in Trizol reagent (Invitrogen, CA, USA) for RNA extraction and cDNA synthesis, while 15 ticks were used for egg laying analysis. RmPAP knockdown was confirmed by qPCR.

### *In vivo* effects of the ingestion of antibodies against RmPAP in partially-fed *R. microplus* females

Partially fed *R*. *microplus* adult females were recovered from calves 20–21 days after larvae infestation. Groups of 30 ticks weighing 25–50 mg were trapped and artificially fed with capillary tubes filled with 50 µL of bovine blood every 2 h for 18 h^44^. The first two feeding cycles contained purified anti-RmPAP or antibodies from non-immunized rabbits (final concentration of 3.5 mg/mL). The biological parameters analyzed were weight gain (initial weight/post-feeding weight), egg production (weight of eggs/initial weight of ticks) and egg hatching (larvae mass/egg mass).

### Statistical analysis

The comparison of RmPAP expression among different tick tissues was performed using the Kruskal-Wallis test with Bonferroni’s multiple comparison post hoc test^45^. The comparison between partially and fully fed ticks was performed using the Mann-Whitney test. RmPAP knockdown was analyzed with Mann-Whitney test and the biological parameters were analyzed using Student’s two-tailed t-test. Analyses were conducted with the Graph Pad Prism 6.0 software (GraphPad Software, Inc.), and differences were considered to be statistically significant when p < 0.05.

## Electronic supplementary material

Supplementary Information
