# Supplementary material for: Novel pseudo-aspartic peptidase from the midgut of the tick Rhipicephalus microplus
Source: Sci Rep. 2019 Jan 24;9:435. doi: 10.1038/s41598-018-36849-4 (PMC6345952; doi:10.1038/s41598-018-36849-4)
Supplement: Supplementary file 1 — Supplementary Information [file 41598_2018_36849_MOESM1_ESM.pdf]

Novel pseudo-aspartic peptidase from the midgut of the tick *Rhipicephalus microplus*.

Lu, S.[1], Parizi, L.F.[2], Torquato, R.J.S.[1], Vaz Junior, I.S.[2,3,4], Tanaka, A.S[1,4].

[1] Department of Biochemistry, Federal University of Sao Paulo (UNIFESP), SP, Brazil.

[2]Center of Biotechnology, Federal University of Rio Grande do Sul (UFRGS), RS, Brazil.

[3] School of Veterinary, Federal University of Rio Grande do Sul (UFRGS), RS, Brazil.

[4] National Institute of Science and Technology in Molecular Entomology (INTC-EM)

Keywords: *Rhipicephalus microplus,* tick, aspartic peptidase, pseudoenzymes

**Corresponding author*: Aparecida Sadae Tanaka, Department of Biochemistry, Escola Paulista de Medicina, UNIFESP. Rua 3 de Maio, 100, 04044-020 São Paulo – SP, Brazil; Tel. +551155764445; E-mail: tanaka.bioq@epm.br

Supplementary data

Material and Methods

Purification of antibodies anti-RmPAP from rabbit sera: Purified recombinant $RmPAP_{WT}$ was adsorbed in CnBr-Activated Sepharose 4B (GE Healthcare) following manufacturer's instructions. Sera from rabbits immunized against $RmPAP_{WT}$ were dialyzed with PBS and applied to the $RmPAP_{WT}$-sepharose resin. Antibodies elution was performed with KCl 0.1 M pH 2.0 in fractions of 1 mL containing 0.1 mL of Tris-HCl 1 M pH 7.5 for pH neutralization. The fractions 18 – 21 were pooled, dialyzed with PBS and concentrated using an Amicon (10 kDa) (Merck) by centrifugation (15 min, 2500 x g at 4°C). Purified antibodies were stored at -20°C prior to use.

Table 1: Primers sequences

	Used in	Restriction sites	Sequence (5' – 3')
RmPAP.FW	pET14b	Xho I	CCGCTCGAGCCACTGGACAACTACAACAATC
RmPAP.RV		Bpu1102	TAGTTATTGCTCAGCCTATTTCGCGATGGCGAAACC
qPAP.FW	qPCR	-	GGATGGAAAATACCAACTGCATAC
qPAP.RV		-	TCTTGTCCGCCAAATATGAAC
qELF1a.FW		-	CGTCTACAAGATTGGTGGCATT
qELF1aRV		-	CTCAGTGGTCAGGTTGGCAG
RmPAP.RNAI.FW	dsRNA	-	GGATCCTAATACGACTCACTATAGGGAATTCTTGGTCATGCTT

RmPAP.RNAI.RV		-	GGATCCTAATACGACTCACTATAGGGGCGGGTCCCCCAATGAA
GFP.RNAi.FW		-	GGATCCTAATACGACTCACTATAGGGTGTTCAATGCTTTGCGAGA
GFP.RNAi.RV		-	GGATCCTAATACGACTCACTATAGGAAAGGGCAGATTGTGTGGAC
RmPAP.MUT.FW	Mutation	-	GCTCGGCCGGCTGACACGGAG
RmPAP.MUT.RV		-	CTCCGTGTCAGCCGGCCGAGC

Table 2: Titration of phages selection with rRmPA

Rounds	Total entry phages	Total output phages	% recovered phages	Enrichment
**1**	$2.09x10^{10}$	$8.55x10^{4}$	$1.03x10^{-5}$	-
**2**	$9.75x10^{8}$	$1.36x10^{5}$	$1.39x10^{-4}$	34.0
**3**	$2.40x10^{8}$	$3.98X10^{4}$	$1.66X10^{-4}$	1.19

```
                 10         20         30         40         50         60
1        ATGCCGATGCTCAGGCTAAGCCTCTTTGTTTTATTGGGATGGGCAGGACCTTGTCTGGCC
1         M  P  M  L  R  L  S  L  F  V  L  L  G  W  A  G  P  C  L  A

                 70         80         90        100        110        120
61       CGTGGCTCAAGTAATGGCCCTGCCGGTCAAGCACTCAAAATGGTGTCGGTACCACTGGAC
21        R  G  S  S  N  G  P  A  G  Q  A  L  K  M  V  S  V  P  L  D

                130        140        150        160        170        180
121      AACTACAACAATCTCACATACATCACAAAGCTCATGCTAGGTACGCCACCGCAGAAATTC
41        N  Y  N  N  L  T  Y  I  T  K  L  M  L  G  T  P  P  Q  K  F

                190        200        210        220        230        240
181      TTGGTCATGCTTGATTCGGCGGGTACAACGTGGGTTCCTGCCTACTACTGCACGCAAGCG
```

```
61          L  V  M  L  D  S  A  G  T  T  W  V  P  A  Y  Y  C  T  Q  A

                  250       260       270       280       290       300
241         GAGCCCTGCTTTGAACACAAGATGTATGACTGGAGCAAGTCGACGACGCAGAGCAGTGAC
81          E  P  C  F  E  H  K  M  Y  D  W  S  K  S  T  T  Q  S  S  D

                  310       320       330       340       350       360
301         TGGGAAGACGGTGCCACACCCTTTGGAAGGGGCAACGTAACCGGCTACGTATTTCGGGAT
101         W  E  D  G  A  T  P  F  G  R  G  N  V  T  G  Y  V  F  R  D

                  370       380       390       400       410       420
361         GCTCATGAGCTCGGCGGCGTGTCCCTCGGAAATCTTCAGTTTGTGAATGGAATAGGCTTC
121         A  H  E  L  G  G  V  S  L  G  N  L  Q  F  V  N  G  I  G  F

                  430       440       450       460       470       480
421         CCCGGGGTGCCCAGCTTCGTGGACAAGCCCTTCGACGGCGTCTTCGGGCTGCGTCTCGGC
141         P  G  V  P  S  F  V  D  K  P  F  D  G  V  F  G  L  R  L  G

                  490       500       510       520       530       540
481         GCAAACTCTACGCTTTACGAAATGGCTAAATCGGGTGTAATCGGCAGTCCAAAGGTGGGA
161         A  N  S  T  L  Y  E  M  A  K  S  G  V  I  G  S  P  K  V  G

                  550       560       570       580       590       600
541         CTTTATTTTAGCAACGACATGACCACTCCAGGGGAAGCCCTCTTCGGAGGTGCAAATGAG
181         L  Y  F  S  N  D  M  T  T  P  G  E  A  L  F  G  G  A  N  E

                  610       620       630       640       650       660
601         CAGCACTACCAAGGTTCAATGACCTTCATGGATGCTCTCGACAGTGCCTTTAAGGTTCAT
201         Q  H  Y  Q  G  S  M  T  F  M  D  A  L  D  S  A  F  K  V  H

                  670       680       690       700       710       720
661         CTAAATGGATATGAAGTGGCTGGTCAGAGAGAAAGGCTACGAGCATCGTGGGCTCGGCCG
221         L  N  G  Y  E  V  A  G  Q  R  E  R  L  R  A  S  W  A  R  P

                  730       740       750       760       770       780
721         GCTCCAACGGAGCCCTTCATTGGAGGACCCGCGAGCGAAATAAAGAAAATCAACACACTG
241         A  P  T  E  P  F  I  G  G  P  A  S  E  I  K  K  I  N  T  L

                  790       800       810       820       830       840
781         CTTGGTGCCAGGAAGATCGAGGATGGAAAATACCAACTGCAGTACGAGCTCCACTGCAAC
261         L  G  A  R  K  I  E  D  G  K  Y  Q  L  Q  Y  E  L  H  C  N

                  850       860       870       880       890       900
841         GCCAGGACTTCCGACTTTACGTTCATATTTGGCGGACAAGAGATCGTCCTGAGGCCAGAA
281         A  R  T  S  D  F  T  F  I  F  G  G  Q  E  I  V  L  R  P  E

                  910       920       930       940       950       960
901         GACTACGTGGTGAAGGTGGAGACAACAACTAAAACTACATGTTACAGCGGCTTCATTGAA
301         D  Y  V  V  K  V  E  T  T  T  K  T  T  C  Y  S  G  F  I  E

                  970       980       990      1000      1010      1020
961         GCGGAAGAGGTGGCGAATGCCAGTTGGCACTTAGGTCTTGTATTCCTCAGACGAGTCTAC
321         A  E  E  V  A  N  A  S  W  H  L  G  L  V  F  L  R  R  V  Y

                 1030      1040      1050      1060      1070      1080
```

```
1021      ACCGTGCTCGAGGCGCCGCTAGAGCCCTCTGGTAATGGACGAGTGGGTTTCGCCATCGCG
341        T  V  L  E  A  P  L  E  P  S  G  N  G  R  V  G  F  A  I  A

1081      AAATAA
361        K  *
```

Sup. Figure 1: Complete nucleotide and translated amino acid sequence of RmPAP amplified from the cDNA library of *Rhipicephalus microplus* midgut. Putative signal peptide is underlined, the Cys residues are in gray boxed and the first Asp residue related to the proteolytic activity is in red.

```
                    10        20
          ....|....|....|....|....|...
WT        RLRASWARPAPTEPFIGGPASEIKKINT
MUT       RLRASWARPADTEPFIGGPASEIKKINT
```

Sup. Figure 2: Alignment of RmPAP Pro^{242} and Asp^{242} amino acid sequence in the mutated region.

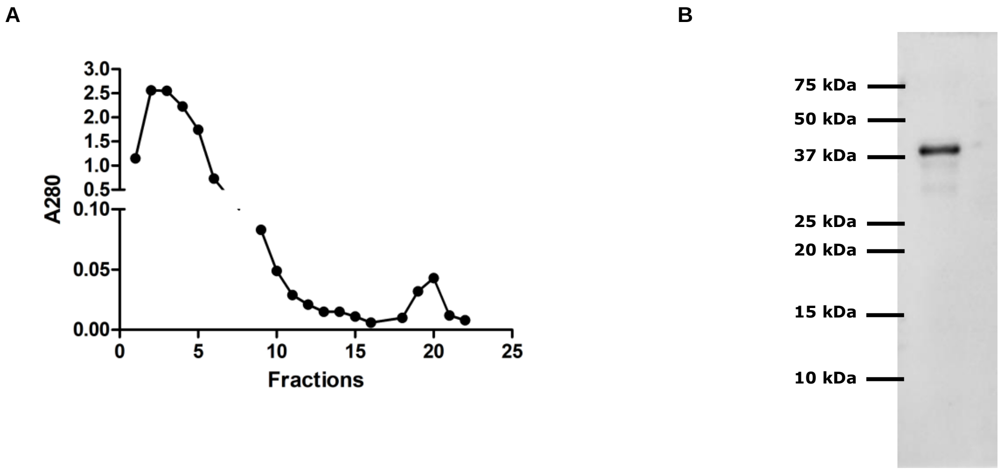

Sup. Figure 3: (A) Purification of anti-RmPAP antibodies using RmPAP-Sepharose CnBr-4B resin. Resin was equilibrated with PBS and 1.0 mL of rabbit sera immunized against rRmPAP was applied to the column, elution was carried with 0.2 M KCl pH 2.0 and pH neutralization was made by the addition of 0.1 mL of 1.0 M Tris-HCl pH 8.0. Fractions 18 – 21 were pooled, concentrated and used for (B) western blot assays with rRmPAP.

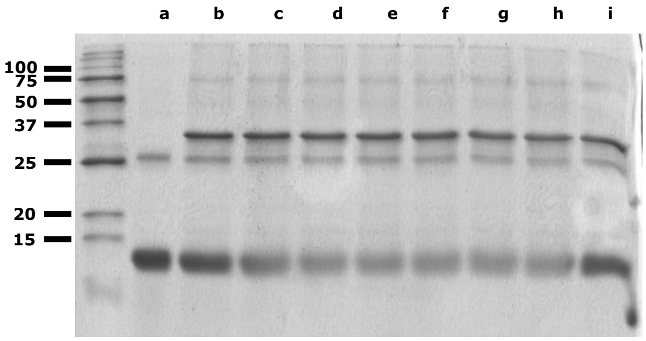

Sup. Figure 4: SDS-PAGE 15% containing the (a) bovine hemoglobin and $RmPA_{MUT}$ with bovine hemoglobin incubated in different pH (b) 2.5, (c), 3.0, (d) 3.5, (e) 4.0, (f) 4.5, (g) 5.0, (h) 5.5 and (i) 6.0.

A B

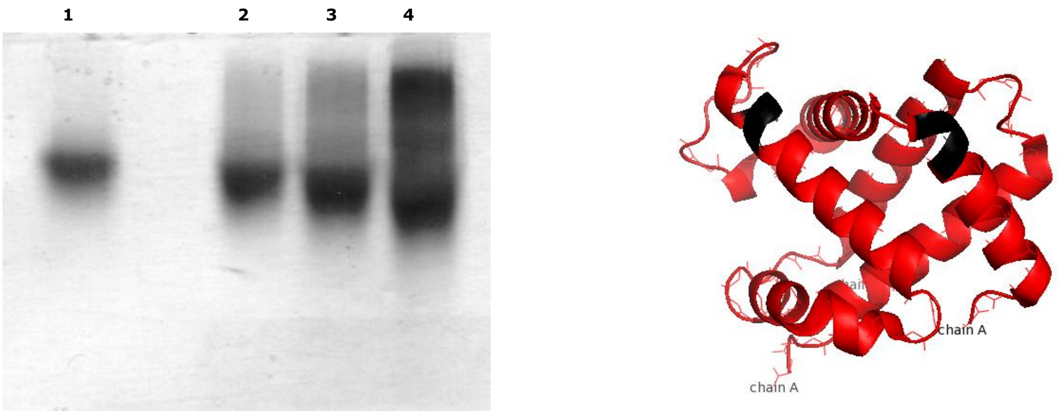

Sup. Figure 5: (A) Native PAGE 12% containing the (1) bovine hemoglobin, (2) bovine hemoglobin:RmPAP$_{MUT}$ (1:1), (3) bovine hemoglobin:RmPAP$_{MUT}$ (1:5), and (4) bovine hemoglobin:RmPAP$_{MUT}$ (1:10). (B) Bovine hemoglobin chain A crystal structure (pdb: 2QSP) with VKG (GKV) regions highlighted in black.
